# Spatial and multilevel analysis of unskilled birth attendance in Chad

**DOI:** 10.1186/s12889-022-13972-6

**Published:** 2022-08-16

**Authors:** Evelyn Acquah, Samuel H. Nyarko, Ebenezer N. K. Boateng, Kwamena Sekyi Dickson, Isaac Yeboah Addo, David Adzrago

**Affiliations:** 1 Centre for Health Policy and Implementation Research, Institute of Health Research, University of Health and Allied Sciences, Ho, Ghana; 2 Department of Epidemiology, Human Genetics, and Environmental Sciences, The University of Texas Health Science Center at Houston, Houston, TX USA; 3 Department of Geography and Regional Planning, University of Cape Coast, Cape Coast, Ghana; 4 Department of Population and Health, University of Cape Coast, Cape Coast, Ghana; 5 Centre for Social Research in Health, The University of New South Wales, Sydney, Australia; 6 Center for Health Promotion and Prevention Research, School of Public Health, The University of Texas Health Science Center at Houston, Houston, TX USA

**Keywords:** Geospatial, Unskilled birth attendance, Multilevel analysis, Chad, Traditional birth attendance, Demographic and Health Surveys (DHS), Social demography, Public health

## Abstract

**Background:**

Unskilled birth attendance is a major public health concern in Sub-Saharan Africa (SSA). Existing studies are hardly focused on the socio-demographic correlates and geospatial distribution of unskilled birth attendance in Chad (a country in SSA), although the country has consistently been identified as having one of the highest prevalence of maternal and neonatal deaths in the world. This study aimed to analyse the socio-demographic correlates and geospatial distribution of unskilled birth attendance in Chad.

**Methods:**

The study is based on the latest Demographic and Health Survey (DHS) data for Chad. A total of 10,745 women aged between 15 and 49 years were included in this study. A multilevel analysis based on logistic regression was conducted to estimate associations of respondents’ socio-demographic characteristics with unskilled birth attendance. Geographic Information System (GIS) mapping tools, including Getis-Ord Gi hotspot analysis tool and geographically weighted regression (GWR) tool, were used to explore areas in Chad with a high prevalence of unskilled birth attendance.

**Results:**

The findings show that unskilled birth attendance was spatially clustered in four Chad departments: Mourtcha, Dar-Tama, Assoungha, and Kimiti, with educational level, occupation, birth desire, birth order, antenatal care, and community literacy identified as the spatial predictors of unskilled birth attendance. Higher educational attainment, higher wealth status, cohabitation, lowest birth order, access to media, not desiring more births, and higher antenatal care visits were associated with lower odds of unskilled birth attendance at the individual level. On the other hand, low community literacy level was associated with higher odds of unskilled birth attendance in Chad whereas the opposite was true for urban residency.

**Conclusions:**

Unskilled birth attendance is spatially clustered in some parts of Chad, and it is associated with various disadvantaged individual and community level factors. When developing interventions for unskilled birth attendance in Chad, concerned international bodies, the Chad government, maternal health advocates, and private stakeholders should consider targeting the high-risk local areas identified in this study.

**Supplementary Information:**

The online version contains supplementary material available at 10.1186/s12889-022-13972-6.

## Background

Despite a substantial global decline in maternal mortality ratio (number of maternal deaths per 100,000 live births) by 38% between 2000 and 2017, several countries in Sub-Saharan Africa (SSA) continue to record high maternal and neonatal deaths as well as significant burdens of preventable pregnancy-related complications [1, 2]. Chad, a country in Central Africa, has consistently been identified over the years as having one of the highest prevalence of maternal and neonatal deaths in the world and is regarded as a very high alert country according to the World Health Organisation’s Fragile States Index [1, 3]. In 2017, for instance, Chad’s maternal mortality ratio was 1,140 per 100,000 live births and the country’s situation was ranked as one of the worst in the world [4].

Utilisation of maternal health services from unskilled birth attendants, defined as “persons who assist a mother during childbirth and who initially acquired their skills by delivering babies themselves or through apprenticeship to other traditional birth attendants” [5], is known to be associated with the adverse maternal health outcomes in Chad, including the incidence of morbidity, disability, and even death [6]. In 2020, it was estimated that less than a quarter (24.3%) of pregnancy and child delivery in Chad were attended by skilled birth attendants with most women utilising services from unskilled birth attendants [7, 8]. A report based on a Multiple Indicator Cluster Survey (MICS) in 2015 also showed that only 22% of child delivery in Chad took place in a health facility with a very low rate (14%) of successful caesarean sections [9].

There is considerable evidence to suggest that most unskilled birth attendants lack the required knowledge and skills to manage complications associated with pregnancy or childbirth, such as haemorrhage, eclampsia, and obstructed labour [10]. Therefore, recognising the spatial distribution of unskilled birth attendance in Chad as well as the factors associated with the high utilisation of services from unskilled birth attendants among women in the country is critical to designing appropriate interventions for improving health outcomes for both women and babies in the country. However, to date, studies on the spatial distribution of unskilled birth attendants and the factors associated with the use of services from unskilled birth attendants in the country are limited. Using the latest nationally representative demographic and health survey data for Chad, this study examined the prevalence, spatial distribution, and the factors associated with the use of services from unskilled birth attendants among women aged 15–49 years in the country. The findings from this study can inform maternal health advocates, health practitioners, policymakers, and other stakeholders in the country to utilise limited health resources judiciously by applying evidence-based interventions to address this significant maternal and neonatal health challenge.

## Methods

### Data source

The study was based on secondary data obtained from the Chad 2014–2015 demographic and health survey (DHS) conducted from October 2014 to April 2015. The survey included a nationally representative sample of 17,719 women aged 15–49 years selected from 17, 233 households. Respondent selection was based on a two-stage stratified cluster sampling procedure. For the first stage, 626 enumeration areas were selected from a list of clusters nationwide [11]. Households were then selected from the complete list of households in each selected cluster during the second stage. For this study, the analysis excluded women who had never given birth within the five years preceding the survey in 2014–2015. The analytic sample, thus, was made up of 10,745 women of reproductive age whose last birth occurred during the five years preceding the survey.

### Study variables and measurements

The outcome variable – unskilled birth attendance – was constructed from the categories used in the DHS concerning the person who assisted with the delivery of the respondent’s last child. In the DHS, the respondents were asked to identify the personnel that assisted them during delivery such as a doctor, nurse or midwife, community health officer or nurse, traditional birth attendant, traditional health volunteer, community or village health volunteer, relative, other, or no one. A binary outcome was constructed by categorising baby deliveries performed by traditional birth attendants, traditional health volunteers, community/village health volunteers, relatives, and others as unskilled birth attendance and the remaining health personnel as skilled birth attendance.

The independent variables of the current study were mainly informed by findings of existing studies on unskilled birth attendance [12–14]. The independent variables considered in the current study are both individual and community level variables measured at the cluster level. The individual-level variables comprised socio-demographic characteristics such as age (15–19, 20–24, 25–29, 30–34, 35–39, 40–44, 45–49), educational level (No formal education, Primary, Secondary, Higher), wealth index (Poorest, Poorer, Middle, Richer, Richest), marital status (Never in a marital union, Married, Cohabitation, Widowed, Divorced, Separated), occupation (Not working, Working), birth order (1, 2–3, 4 +), media exposure (No, Yes), desire for birth (Then, later, no more), and antenatal care visits (Less than 4, 4 or more).

Community variables were computed at the primary sampling unit level. Community socioeconomic status was computed from occupation, wealth, and education of study participants who resided in each sampled cluster as all community variables are for only women. Principal component analyses were applied based on women who were unemployed, uneducated, or from poor households. A standardised rating was derived with an average rating (zero) and standard deviation [15]. The rankings were then segregated into tertiles where the lower scores (tertile 1) denote high socioeconomic status, and average scores (tertile 2) as moderate socioeconomic status while the higher scores (tertile 3) denote lower socioeconomic status.

Respondents who attended higher than secondary school were considered literate while all other respondents were given a sentence to read and were considered literate if they could read all or part of the sentence. Therefore, if respondents had higher than secondary education or had no school/primary/secondary education but could read a whole sentence, it was considered as high literacy. Medium literacy represented respondents who had no school/primary/secondary education and could read part of the sentence. Low literacy comprised respondents who had no school/primary/secondary education and could not read at all. Thus, socioeconomic status and literacy level were measured as low, moderate, and high at the primary sampling unit level (cluster) while place of residence was measured as urban or rural. The explanatory variables of the current study were mainly informed by findings of existing studies on unskilled birth attendance [12–14].

### Analytical strategy

#### Descriptive and logistic regression analyses

Two levels of analysis were conducted. A descriptive analysis was performed by calculating the proportion of respondents’ socio-demographic characteristics. Multilevel logistic regression analysis was conducted to estimate associations of respondents’ socio-demographic characteristics with unskilled birth attendance. The Model 1 (null model) was estimated to establish whether there is a significant variance in unskilled birth attendance at the cluster level to justify the multilevel analysis, while Model 2 comprises the fixed effects analysis of individual-level socio-demographic factors and assumes that these socio-demographic factors have fixed or constant association with unskilled birth attendance across all the primary sampling units. Model 3 estimated the effects of the community-level variables and assumed that the effects may vary across the primary sampling units. Model 4 contains the full model (Models 1, 2, and 3) and the mainstay of the analysis. Adjusted odds ratios and 95% confidence intervals were calculated for the variables. All analyses were conducted with Stata software (Version 14) and the results were weighted to cater for potential over-sampling and under-sampling.

#### Geospatial analysis

Regarding the spatial analysis, the coordinates of the surveyed respondents were obtained from the DHS website. These data were projected to UTM Zone 33 N to aid the spatial analysis. Relying on just the surveyed point was insufficient, so Chad’s departments’ shapefile was obtained from the Humanitarian Data Exchange for the spatial analysis [16]. The departments’ shapefile was also projected to UTM Zone 33 N. This dataset had 70 departments, but 55 departments were found to have had respondents sampled for the survey. Therefore, the 15 additional departments were excluded from the analysis.

As part of the dependent variable’s data processing, birth attendance was coded 0 for skilled birth attendance and 1 for unskilled birth attendance. The independent variables were in their original categories, but proportions were generated from the lower categories at each sub-regional level. Therefore, the extracted surveyed data was linked with its corresponding coordinates. These data were then merged with the sub-regional shapefile using the join tool. In joining, the average computation system was adopted to estimate averages of unskilled birth attendance at the sub-regional level (departments). After obtaining the sub-regional level estimate of unskilled birth attendance, the independent variables’ proportions were added to asssist with the computation of the geographically weighted regression (GWR).

The actual data analysis began with using the spatial autocorrelation tool (Moran’s I) to assess the distribution of unskilled birth attendance in Chad. The spatial autocorrelation assesses the distribution of a phenomenon being studied; thus, either random, dispersed, or clustered. The output does not show the exact sub-regional distribution of unskilled birth attendance. Therefore, the Getis-Ord Gi hotspot analysis tool was used to examine areas that had a higher tendency of experiencing unskilled birth attendance. A limitation of the hotspot tool is that it considers areas with high values of the dependent variables to create hot and cold spots. However, it is advisable to use the Anselin Local Moran’s I cluster and outlier analysis tool for policy implications to explore other areas that may not be identified as a hotspot but have a high incidence of the phenomenon being studied. The final analysis was to determine the spatial explanation of the independent variables by running the GWR. GWR is a spatial regression technique that focuses on the spatial differentiation in the explanatory power of independent variables. This is done by estimating separate regression equations between the dependent and independent variables to every feature in the dataset. Before running the GWR, significant independent variables were identified using the exploratory regression analysis. This regression method was adopted because it assesses all possible combinations of the independent variables that best explain the dependent variable. Thus, it looks for models that meet all of the requirements and assumptions of the OLS method. The first model of the fifth category was accepted since it had the criteria [AICc = -52.19, JB = 0.08, K(BP)0.02, VIF = 1.71, SA = 0.46] that contributed to the highest adjusted R^2^ (0.32). This revealed the best combination of independent variables that are significant predictors of unskilled birth attendance in Chad. This allowed the use of the identified independent variables that were used for the GWR. In conducting the GWR, the identified independent variables found to be significant predictors of unskilled birth attendance in Chad were used as the explanatory variables with kernel type being fixed as well as bandwidth method using the AICc. The obtained adjusted R^2^ was about 0.309 percent which implies that the results explain about 31% of the entire data. All the data processing and analyses were conducted in ArcGIS version 10.7.

#### Model fit and specifications

The Likelihood Ratio (LR) test was used to evaluate the fitness of all the models. Before fitting the models, the presence of multicollinearity between the independent variables was evaluated. The variance inflation factor (VIF) test found the absence of high multicollinearity between the variables (Mean VIF = 1.94).

## Results

### Socio-demographic characteristics and prevalence of unskilled birth attendants

The prevalence of unskilled birth attendants was 61.5 percent. More than half of the women who gave birth had no formal education (65. 1%), were married (83.6%), desired birth (81.3%), had low socioeconomic status (65.2%), and were residents of rural areas (80%) while few had higher education (0.6%), never been in a marital union (1.2%), desired for birth later (14.5%) had moderate socioeconomic status (4.3%) and were residents of urban areas (19.8%) (see Table 1).

**Table 1:** Background characteristics and prevalence of unskilled birth attendants

Variables	Frequency (*N* = 10,745)	Percentage	Proportion of unskilled birth attendants	X^2^ (*p*-value)
**Individual variables**				
*Age*				27 (0.000)
15–19	1152	10.7	58.1	
20–24	2365	22.0	60.4	
25–29	2822	26.3	62.3	
30–34	2085	19.4	61.9	
35–39	1411	13.1	63.9	
40–44	712	6.6	59.6	
45–49	197	1.9	59.6	
*Educational level*				1.1e + 03 (0.000)
No formal education	7000	65.1	71.3	
Primary	2568	23.9	51.3	
Secondary	1113	10.4	27.2	
Higher	64	0.6	1.7	
*Wealth index*				1.3e + 03 (0.000)
Poorest	2215	20.6	71.1	
Poorer	2307	21.5	69.3	
Middle	2171	20.2	70.5	
Richer	2164	20.1	65.6	
Richest	1888	17.6	25.8	
*Marital status*				204 (0.000)
Never in a marital union	135	1.2	28.8	
Married	8984	83.6	64.4	
Cohabitation	900	8.4	46.2	
Widowed	177	1.7	54.5	
Divorced	229	2.1	48.4	
Separated	320	3.0	52.9	
*Occupation*				10 (0.001)
Not working	5053	47.0	62.7	
Working	5692	53.0	60.5	
*Birth order*				57 (0.000)
1	1556	14.5	52.8	
2–3	2962	27.6	62.6	
4 +	6227	57.9	63.2	
*Media*				945 (0.000)
No	7741	72.0	70.2	
Yes	3004	28.0	39.1	
*Desire for birth*				55 (0.000)
Then	8734	81.3	63.0	
Later	791	7.4	61.9	
No more	1220	11.3	50.6	
*Antenatal care visits*				937 (0.000)
Less than 4	7204	67.0	71.2	
4 or more	3541	33.0	41.9	
**Community variables**				
*Socioeconomic status*				776 (0.000)
Low	7004	65.2	70.7	
Moderate	466	4.3	56.7	
High	3275	30.5	42.6	
*Literacy level*				1.3e + 03 (0.000)
Low	4539	42.3	79.0	
Moderate	1917	17.8	59.7	
High	4289	39.9	43.9	
*Place of residence*				1.2e + 03 (0.000)
Urban	2124	19.8	29.8	
Rural	8621	80.2	61.5	
**Total**	10,745		61.5	

Seven in ten women with no formal education (71.3%), poorest wealth status (71,1%), no media access (70.2%), less than four antenatal visits (71.2%), low socioeconomic status (70.7%), and low literacy levels (79.0%) utilised the services of unskilled birth attendants during delivery (see Table 1). The proportion of women utilising the services of unskilled birth attendants was also high among women aged 35–39 (63.9%), married (64.4%), not working (62.7%), desired birth (63.0%), and of rural residence (61.5%) (see Table 1).

### Multilevel analysis of unskilled birth attendance among women in Chad

The logistic regression of unskilled birth attendance is presented in Table 2. The results showed significant associations between utilisation of services of unskilled birth attendants during delivery and educational level, wealth status, marital status, birth order, access to media, desire for birth, antenatal visits, literacy level, and place of residence. Association between formal education and unskilled birth attendance was significant for all levels of formal education. All wealth indices were significantly associated with the utilisation of services of unskilled birth attendants.

**Table 2:** Multilevel analysis of unskilled birth attendance

Variables	Model 1	Model 2	Model 3	Model 4
	Odds Ratio (95% Confidence interval)	Odds Ratio (95% Confidence interval)	Odds Ratio (95% Confidence interval)	Odds Ratio (95% Confidence interval)
**Individual variables**				
*Age*				
15–19		Ref		Ref
20–24		0.97(0.78, 1.21)		0.98(0.79,1.22)
25–29		0.93(0.73, 1.18)		0.93(0.73,1.18)
30–34		0.94(0.72, 1.23)		0.95(0.73,1.25)
35–39		1.02(0.77, 1.37)		1.03(0.76,1.37)
40–44		0.83(0.60, 1.16)		0.84(0.60,1.17)
45–49		1.09(0.67, 1.76)		1.12(0.69,1.80)
*Educational level*				
No formal education		Ref		Ref
Primary		0.70***(0.61,0.81)		0.76***(0.65,0.88)
Secondary		0.37***(0.29,0.46)		0.43***(0.34,0.53)
Higher		0.05**(0.01,0.37)		0.06**(0.01,0.48)
*Wealth index*				
Poorest		Ref		Ref
Poorer		0.77**(0.65,0.92)		0.74**(0.62,0.88)
Middle		0.87(0.73,1.04)		0.83*(0.69,0.99)
Richer		0.65***(0.54,0.78)		0.66***(0.55,0.79)
Richest		0.28***(0.22,0.36)		0.52***(0.40,0.68)
*Marital status*				
Never in a marital union		0.65(0.35,1.19)		0.66(0.36,1.21)
Married		Ref		Ref
Cohabitation		0.52***(0.42,0.65)		0.54***(0.43,0.67)
Widowed		0.81(0.54,0.78)		0.89(0.59,1.34)
Divorced		0.88(0.62,1.26)		0.91(0.63,1.29)
Separated		0.78(0.56,1.09)		0.83(0.60,1.16)
*Occupation*				
Not working		1.04(0.91,1.17)		1.03(0.91,1.17)
Working		Ref		Ref
*Birth order*				
1		0.74**(0.58,0.92)		0.73**(0.58,0.92)
2–3		1.10(0.94,1.29)		1.10(0.94,1.29)
4 +		Ref		Ref
*Media*				
No		Ref		Ref
Yes		0.68***(0.60,0.79)		0.75***(0.65,0.86)
*Desire for birth*				
Then		Ref		Ref
Later		1.11(0.89,1.38)		1.12(0.89,1.40)
No more		0.76*(0.63,0.91)		0.79*(0.65, 0.95)
*Antenatal care visits*				
Less than 4		Ref		Ref
4 or more		0.41***(0.37,0.47)		0.44***(0.39,0.49)
**Community variables**				
*Socioeconomic status*				
Low			Ref	Ref
Moderate			0.77(0.39,1.54)	0.76(0.40,1.44)
High			0.67**(0.48,0.94)	0.83(0.60,1.16)
*Literacy level*				
Low			5.68***(4.23,7.64)	3.03***(2.26,4.06)
Moderate			1.84***(1.25,2.71)	1.34(0.93,1.93)
High			Ref	Ref
*Place of residence*				
Urban			0.25***(0.17,0.37)	0.35***(0.24,0.52)
Rural			Ref	Ref
**Random effect result**				
PSU variance (95% CI)	3.80(3.23, 4.45)	1.81(1.50, 2.16)	1.80(1.52, 2.14)	1.56(1.31, 1.85)
ICC	0.54	0.35	0.35	0.32
LR Test	χ^2^ = 2928.09 p = 0.0000	χ^2^ = 1178.42 p = 0.0000	χ^2^ = 1450.46 p = 0.0000	χ^2^ = 1140.80 p = 0.0000
Wald Chi-square		638.76	417.57	821.68
Model fitness				
Log-likelihood	-5416.36	-5087.58	-5237.75	-5009.66
BIC	10,851.29	10,425.78	10,540.47	10,316.35
AIC	10,836.72	10,229.16	10,489.49	10,083.32
N	10,745	10,745	10,745	10,745

*AIC* Akaike’s information criterion, *ICC* intra-cluster correlation

Ref reference category *p < 0.05 **p < 0.01 ***p < 0.001

In the fourth model, the results revealed that women with higher levels of formal education were less likely (AOR = 0.06, CI = 0.01, 0.48) to utilise the services of unskilled birth attendants during delivery compared to those with no formal education. Those within the richest wealth status were less likely (AOR = 0.52, CI = 0.40, 0.68) to utilise the services of unskilled birth attendants during delivery compared to those with the poorest wealth status. The lesser likelihood was applicable for all wealth indices above the poorest wealth status. Women who were cohabiting were less likely (AOR = 0.54, CI = 0.43, 0.67) to utilise the services of unskilled birth attendants during delivery compared to those who were married. Women with access to media had a lesser likelihood (AOR = 0.75, C1 = 0.65, 0.86) of utilising the services of unskilled birth attendants during delivery compared to those with no access to the media. The likelihood of utilising the services of unskilled birth attendants was lesser (AOR = 0.44, CI = 0.39, 0.49) among women who made 4 or more antenatal visits compared to women who made less than 4 visits (see Table 2).

Those who desired birth no more were less likely (AOR = 0.75, CI = 0.65,0.86) to utilise the services of unskilled birth attendants during delivery compared to those who then desired for birth. The likelihood of utilising the services of unskilled birth attendants was higher (AOR = 1.10 CI = 0.94,1.29) among women who had 2–3 birth order compared to women who had 4 or more. Those with low community literacy levels were more likely (AOR = 3.03, CI = 2.26,4.06) to utilise the services of unskilled birth attendants during delivery compared to those with high literacy levels. Likelihood of utilising the services of unskilled birth attendants was less (AOR = 0.35, CI = 0.24,0.52) among women of urban residence compared to those of rural residence (see Table 2).

### Random effects of clusters (measures of variations) results

Concerning the clustering of PSUs, the empty model (Model 1) demonstrated low variance in the likelihood of delivery assisted by unskilled birth attendants (σ^2^ = 3.80, 95% CI = 3.23, 4.45) (See Table 2). The empty model also revealed that inter-cluster variation of the characteristics accounts for 54% of the overall variance in unskilled birth attendance and delivery assisted by unskilled birth attendants declined (ICC = 0.54). The likelihood ratio of delivery assisted by unskilled birth attendants decreased in model 3 (σ^2^ = 1.80, 95% percent CI = 1.52, 2.14). However, due to inter-cluster heterogeneity in the features, the total variance in deliveries aided by unskilled birth attendants decreased (32%). This suggests that disparities in delivery assisted by unskilled birth attendants are partly due to unaccounted community-level characteristics, as illustrated in Model 4. (See Table 2).

### Spatial distribution results

**Fig. 1:**
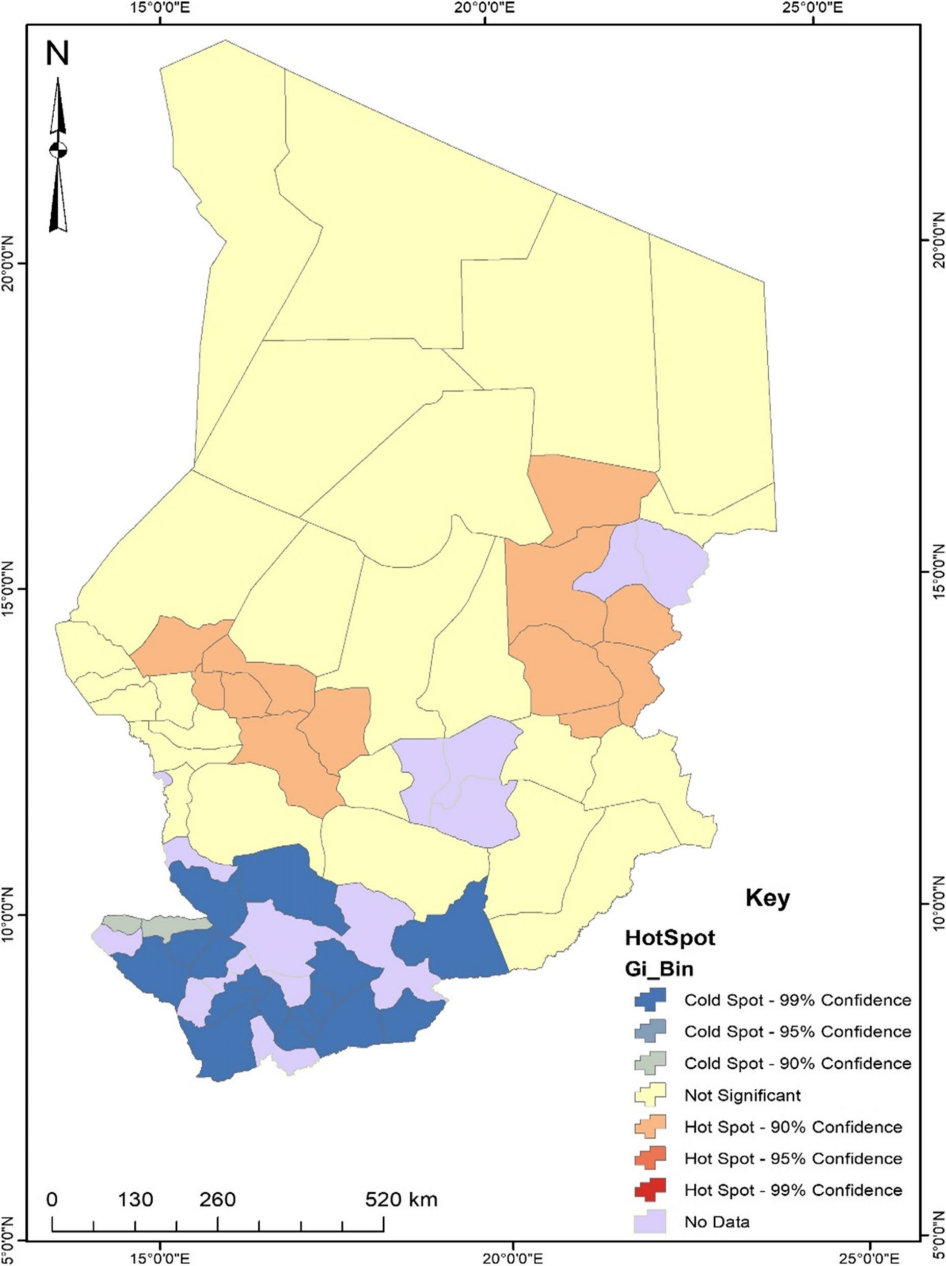
Hotspot analysis of unskilled birth attendance in Chad. Source: Authors’ construct (2021)

Based on the Moran’s I result (Additional file 1), the spatial distribution of unskilled birth attendance in Chad was clustered. This means that the spatial distribution of unskilled birth attendance in Chad was not randomised and can be found around a particular area. Therefore, the study used the Getis-Ord Gi hotspot analysis to visualise the distribution of unskilled birth attendance in Chad (see Fig. 1).

In reference to Fig. 1, areas in the south of Chad were found to be cold spots; thus, in those areas, there was 99% less chance of finding women who utilise the services of unskilled birth attendants. Therefore, Lac Iro, Grande Sido, Barh-Sara, Mandoul Oriental, Mandoul Occidental, Kouh Est, Kouh Ouest, La Nya, Ngourkosso, Lac Wey, Monts de Lam, Mayo-Dallah, Tandjile Ouest, Kabbia, Mayo-Boneye, and Loug-Chari were specifically found to have low utilisation of services from unskilled birth attendants compared to other areas. On the contrary, departments such as Mourtcha, Biltine, Ouara, Dar-Tama, Assoungha, Abdi, Kanem, Wadi Bissam, Barh-El-Gazel Sud, Barh-El-Gazel Ouest, Dababa, and Fitri were found to be the hotspots of unskilled birth attendance in Chad. This implies that women from these areas had high utilisation of services from unskilled birth attendants. However, these areas (departments) were clustered around the same region of the country. This validates the Moran’s I results of showing that the distribution of unskilled birth attendance is clustered in some parts of the country. From Fig. 1, women in those areas had a 90% chance of utilising the services of skilled birth attendants. It is important to however acknowledge that departments of Lac Iro, Grande Sido, Barh-Sara, Mandoul Oriental, Mandoul Occidental, Kouh Est, Kouh Ouest, La Nya, Ngourkosso, Lac Wey, Monts de Lam, Mayo-Dallah, Tandjile Ouest, Kabbia, Mayo-Boneye, and Loug-Chari were located in provinces that were close (within 8-13 km) to healthcare centres compared to departments of Mourtcha, Biltine, Ouara, Dar-Tama, Assoungha, Abdi, Kanem, Wadi Bissam, Barh-El-Gazel Sud, Barh-El-Gazel Ouest, Dababa, and Fitri where households were about 14 – 55 km away from healthcare centres. To overcome the limitations of the hotspot analysis and develop interventions to curb the issue of unskilled birth attendance, cluster and outlier analysis was conducted.

**Fig. 2:**
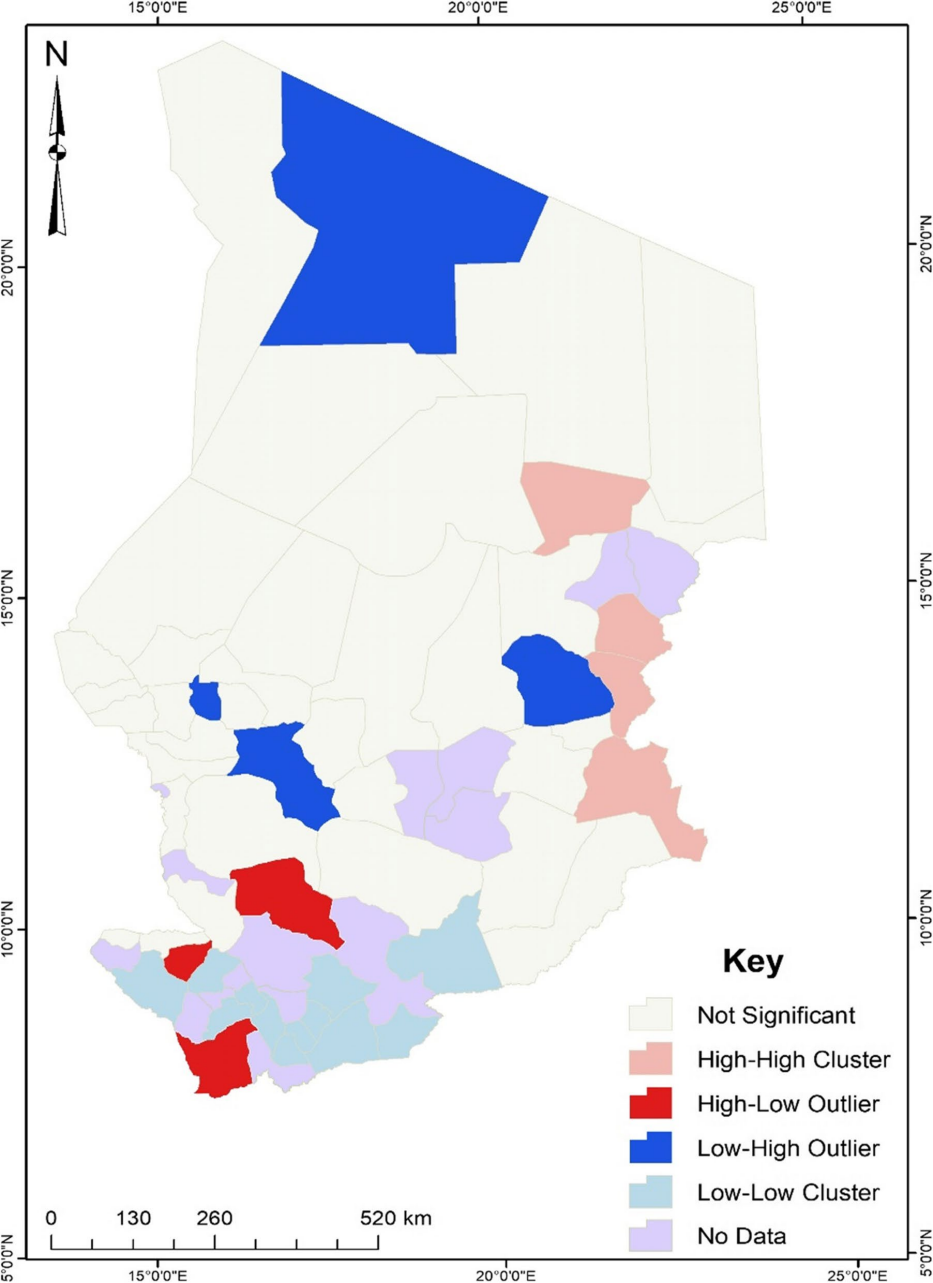
Clusters and outliers of unskilled birth attendance in Chad. Source: Authors’ construct (2021)

Results from the Anselin Local Moran’s I cluster and outlier analysis (see Fig. 2) revealed that four departments had a high incidence of unskilled birth attendance and shared boundaries with departments with a high incidence of unskilled birth attendance. These departments were Mourtcha, Dar-Tama, Assoungha, and Kimiti. Three departments were found to have a high incidence of unskilled birth attendance but shared boundaries with departments with a low incidence of unskilled birth attendance. These departments were Loug-Chari, Kabbia, and Monts de Lam.

As displayed in Fig. 2, four departments were found to have low incidence but shared boundaries with areas with a high incidence of unskilled birth attendance. These departments were Tibesti Est, Ouara, Dababa, and Wadi Bissam. Also, 12 departments were found to be low and surrounded by departments with a low incidence of unskilled birth attendance. These sub-regions were Mayo-Dallah, Tandjile Ouest, Lac Wey, Ngourkosso, La Nya, Kouh Ouest, Kouh Est, Mandoul Occidental, Mandoul Oriental, Barh-Sara, Grande Sido and Lac Iro.

Before conducting the GWR, the exploratory regression analysis revealed that independent variables such as educational level, occupation, birth desire, birth order, antenatal care, and community literacy were significant spatial predictors of unskilled birth attendance in Chad. These predictors were then used for the GWR analysis. According to the regression model fit, the adjusted R^2^ value explains about 31% with a Sigma value of 0.14 and an AICc value of -49.48. The maximum coefficient p-value was pegged at 0.05 with a VIF value of 7.6 and a Jarque–Bera p-value of 0.10. To understand the maps generated out of the regression, areas displayed in red show a robust predictive power, whereas areas displayed in blue indicate a low predictive power of the independent variable in terms of the occurrence of unskilled birth attendance.

**Fig. 3:**
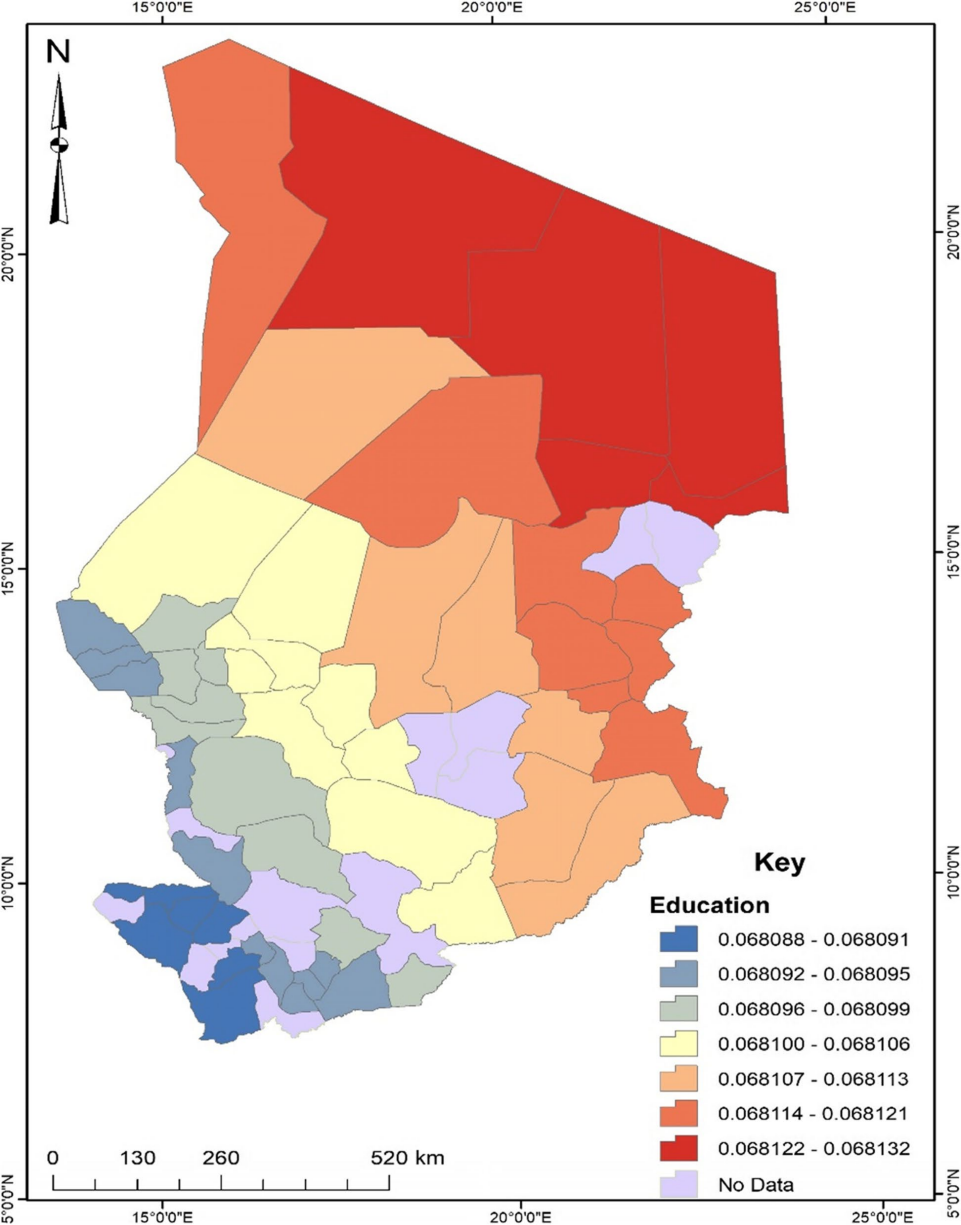
Education GWR coefficient for predicting unskilled birth attendance in Chad. Source: Authors’ construct (2021)

As shown in Fig. 3, the proportion of women with no formal education who were likely to experience unskilled birth attendance can be found in the northern and eastern parts of Chad. This implies that women with no formal education found in the departments located in the northern and eastern part of Chad stand a higher chance of experiencing unskilled birth attendance. The likelihood of unskilled birth attendance occurring among women without formal education was about 6.8% higher than their counterparts.

**Fig. 4:**
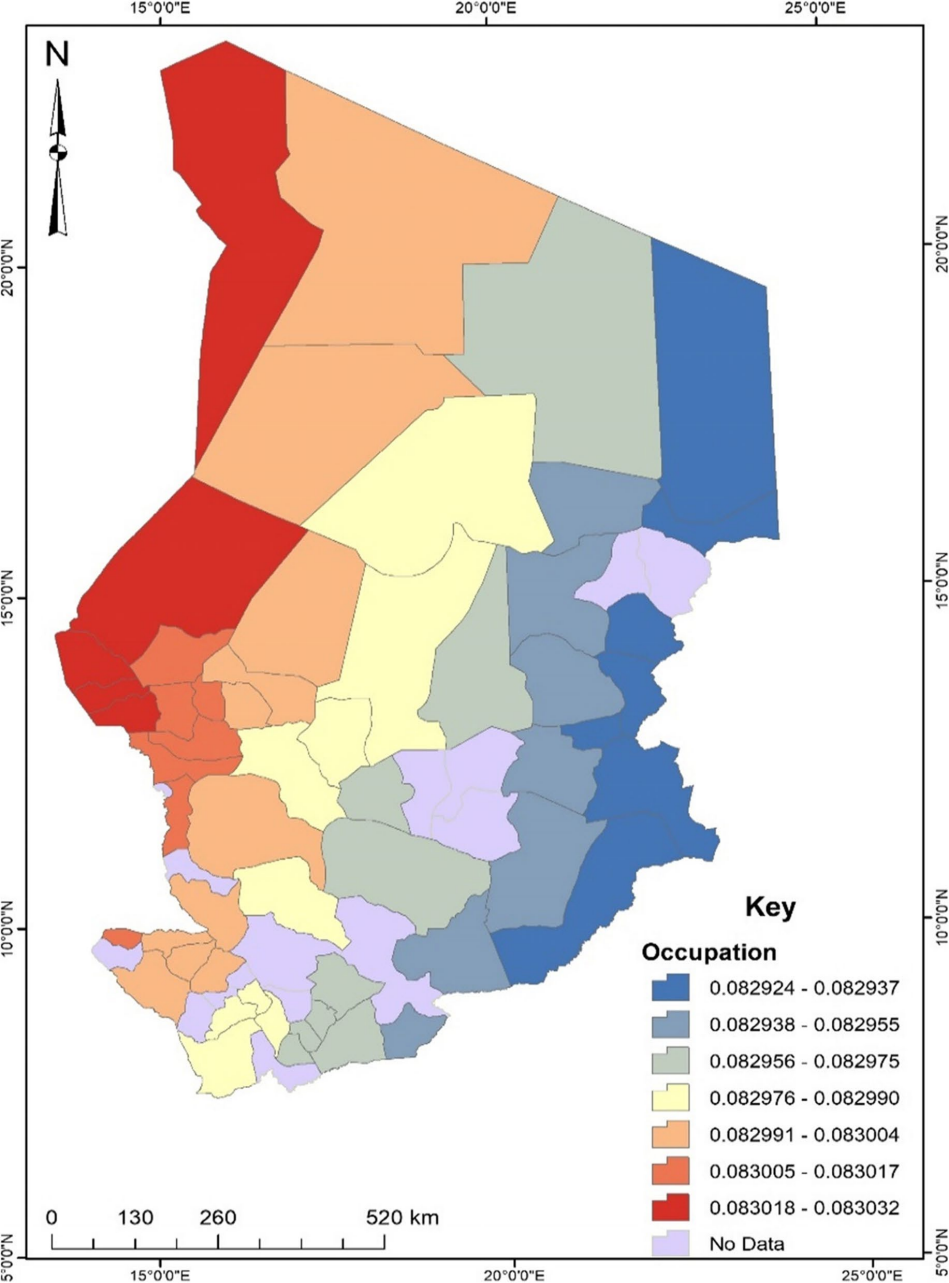
Occupation GWR coefficient for predicting unskilled birth attendance in Chad. Source: Authors’ construct (2021)

Regarding occupation, unemployed women were about 8.3% more likely to experience unskilled birth attendance than their counterparts. Spatially, the proportion of unemployed women being a predictor of unskilled birth attendance is likely to occur in the western part of Chad (see Fig. 4).

**Fig. 5:**
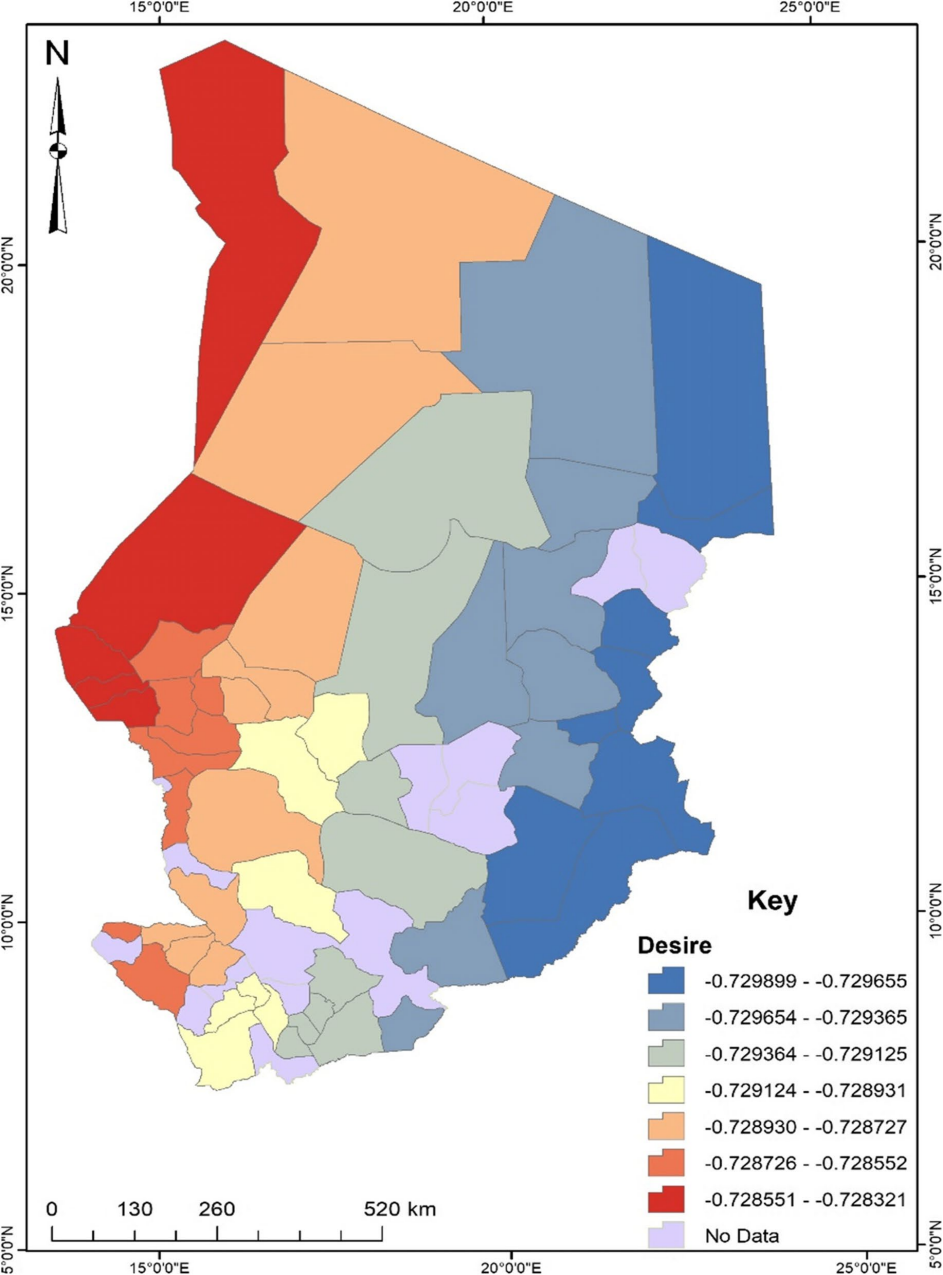
Desire GWR coefficient for predicting unskilled birth attendance in Chad. Source: Authors’ construct (2021)

Also, women with no desire to have more children were more likely to experience unskilled birth attendance especially in the western parts of Chad (see Fig. 5). A unit decrease in no more desire to have more children would result in about a 72% increase in the experience of unskilled birth attendance in Chad.

**Fig. 6:**
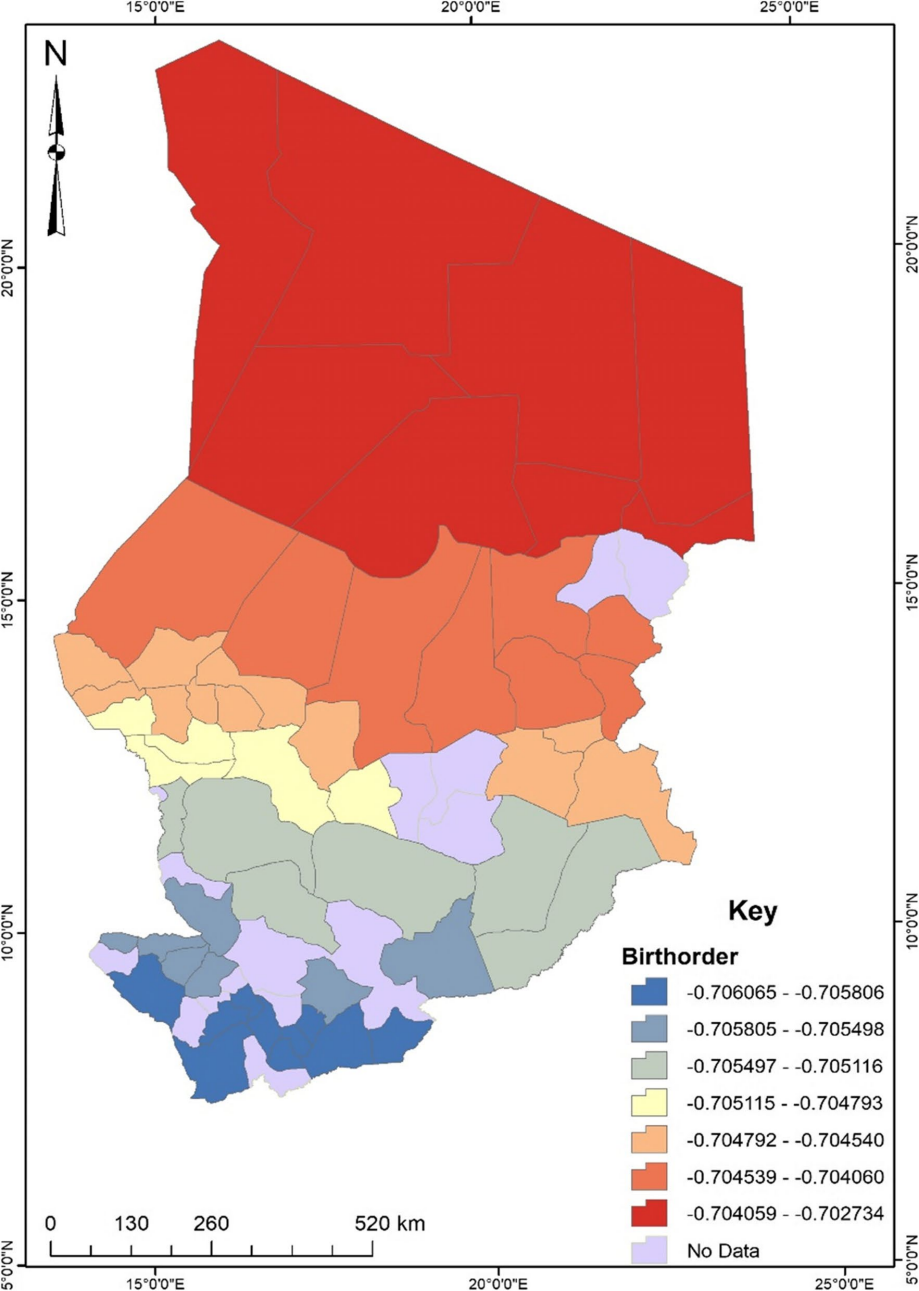
Birth order GWR coefficient for predicting unskilled birth attendance in Chad. Source: Authors’ construct (2021)

The women with one birth order were less likely to experience unskilled birth attendance in the northern part of Chad (see Fig. 6). This implies that the women were less likely to experience unskilled birth attendance in Chad as their birth order decreases. A unit decrease in birth order would result in about a 70% increase in unskilled birth attendance.

**Fig. 7:**
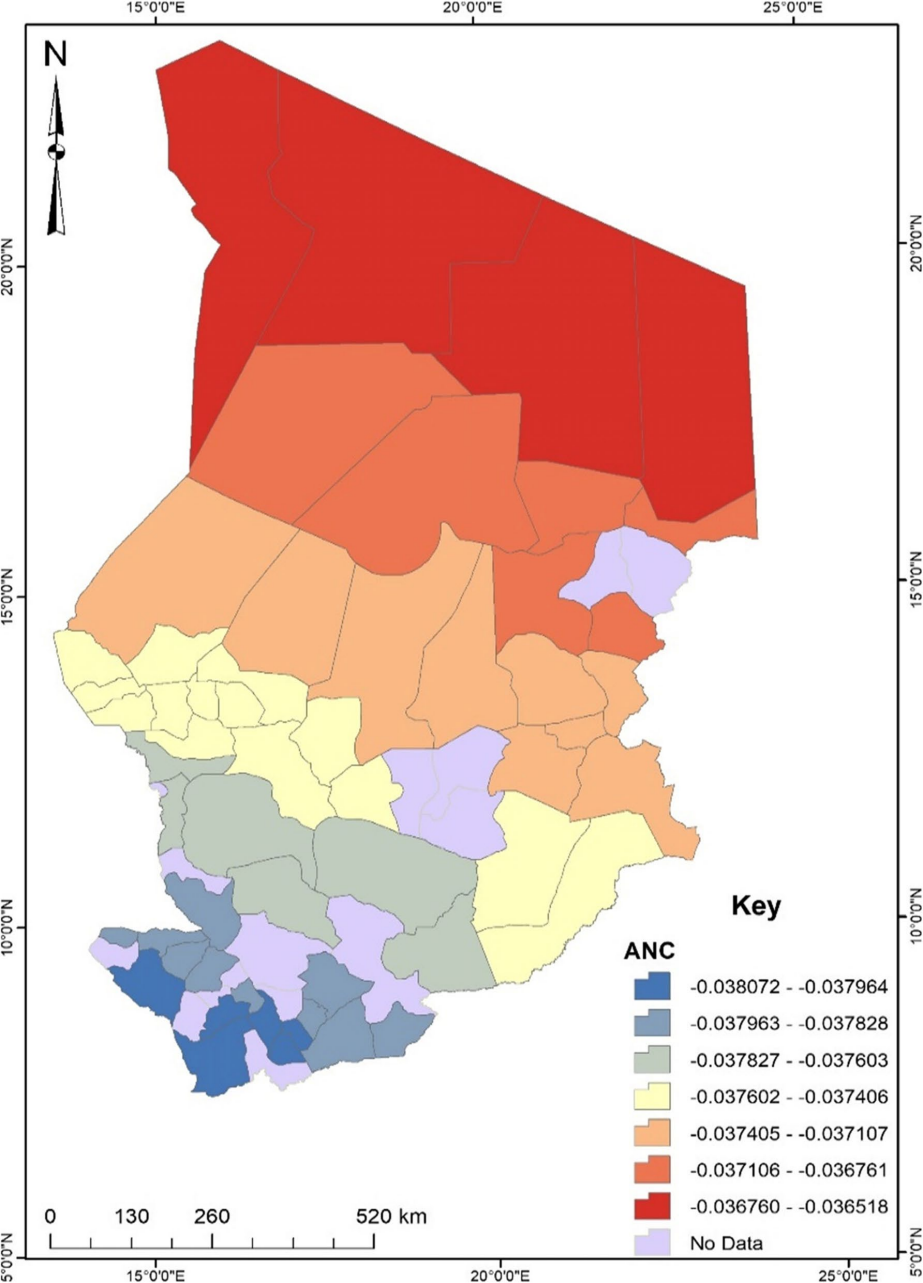
ANC GWR coefficient for predicting unskilled birth attendance in Chad. Source: Authors’ construct (2021)

Moreover, women who did not attend antenatal care were more likely to experience unskilled birth attendance in Chad. Spatially, Fig. 7 shows that women who did not attend antenatal care in the northern part of Chad were more likely to experience unskilled birth attendance. A unit decrease in the attainment of antenatal care would result in about a 3% increase in the experience of unskilled birth attendance in Chad.

**Fig. 8:**
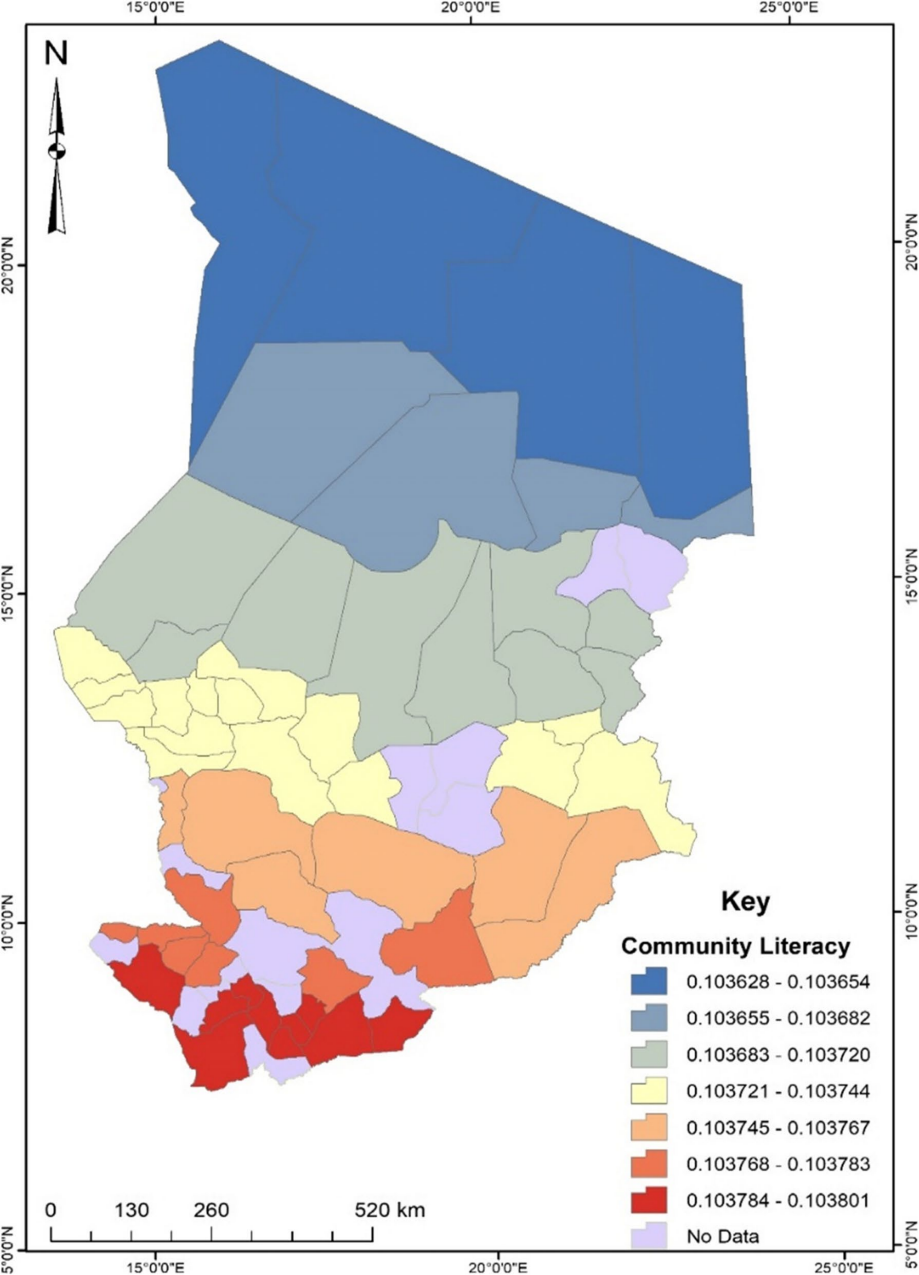
Community literacy GWR coefficient for predicting unskilled birth attendance in Chad. Source: Authors’ construct (2021)

Finally, the proportion of women with low community literacy serves as a high predictor of unskilled birth attendance in the southern part of Chad (see Fig. 8). As their community literacy decreases, their unskilled birth attendance increases by about 10%.

## Discussion

This study aimed to understand the geospatial distribution and prevalence of unskilled birth attendance in relation to different socio-demographic characteristics of women in Chad. While some women were less likely to seek services from unskilled birth attendants and experience its associated consequences, the opposite was true for others. Among the women, having no formal education, being in the poorest wealth index, having at least a fourth child, not exposed to the media, having past desire for birth, having less than four antenatal care visits, living in community areas with low socioeconomic status (i.e., occupation, wealth) or low/moderate level of literacy, and living in rural areas were associated with a higher likelihood of obtaining services from unskilled birth attendants. Our findings are consistent with a previous study that focused on skilled birth attendance in SSA [7] and also confirm the assumption that a significant proportion of women in Chad are lacking adequate formal education, having high unemployment rates, or having limited media exposure that may be affecting their knowledge and financial capabilities to seek skilled maternal health services, hence contributing to high burdens of maternal morbidity and deaths [7, 8].

Findings from this study also demonstrate that between and within communities in Chad, there are significant wealth-related inequalities associated with seeking the services of unskilled birth attendants. The disparities between communities explained about 32% of the differences in seeking the services of unskilled birth attendants, suggesting that about 68% of the differences in seeking the services of unskilled birth attendants can be attributed to differences within the community. Similarly, earlier studies indicated that there are disparities in seeking services from skilled birth attendants in SSA countries, including Chad, where there are poorer socioeconomic conditions, such as limited access to health facilities and wealth [7, 17, 18]. Communities with these poor socioeconomic conditions have limited health resources, including skilled birth attendants, that potentially predispose them to obtain services from unskilled birth attendants. Results from our spatial analysis revealed evidence of spatial clustering of unskilled birth attendants in some parts of Chad. The hotspot analysis used to visualise the distribution of unskilled birth attendants showed that the prevalence of unskilled birth attendants is highest in all parts except the southern part of Chad (i.e., it is lowest in the southern part). As shown by our cluster and outlier analysis, 4 of the 55 sub-regions or departments had a high incidence of unskilled birth attendance and shared boundaries with departments with a low incidence of unskilled birth attendance. A possible explanation for this finding is that the incidence of unskilled birth attendance in the four sub-regions might not be related to the incidence in their neighbouring sub-regions due to better socioeconomic conditions (e.g., quality health facilities and services, wealth, and higher levels of formal education) in those neighbouring sub-regions. Potential effective maternal health interventions, such as providing affordable services from skilled birth attendants targeting the sub-regions of Chad where services of unskilled birth attendants are more clustered may eventually reduce spatial inequalities in seeking maternal health services and improve access to services of skilled birth attendants. Additionally, improving the socioeconomic conditions (e.g., health facilities, wealth, and employment) of the sub-regions may increase access to healthcare and enhance maternal health in general.

Studies found that socioeconomic conditions with geographic inequalities have significant effects on health outcomes and behaviours, such as seeking services of unskilled birth attendants in SSA countries including Chad [7, 12, 19]. Our geographically weighted regression (GWR) analysis showed similar findings, indicating that some regional contextual factors were associated with the prevalence of unskilled birth attendance among women in Chad. Women with no formal education in sub-regions or departments of the northern and eastern parts of Chad were about 6.8% more likely to experience or seek the services of unskilled birth attendants. A possible reason is that women with no formal education in those areas may have limited knowledge about the possible consequences of unskilled birth attendance, or where to seek services of skilled birth attendants [7, 12]. Occupational or employment disparities also play a significant role in the prevalence of unskilled birth attendance among women in Chad. Women residing in the western part with high proportions of unemployment were about 8.3% more likely to experience or seek the services of unskilled birth attendants. Perhaps, this economic disadvantage may have compounded the women’s inability to seek services from skilled birth attendants, thereby making them seek services from unskilled birth attendants that may be financially cheaper but have higher risk of adverse health consequences [8]. The Boko Haram attacks in the western parts such as the Lac region of Chad might have also affected the women’s visits to health centres as well as seeking the services of skilled birth attendants. Congruently, unskilled birth attendance was about 72% more likely to occur among women who no longer desired to have a child or children in the western part of Chad. Although the women in the northern part of the country with a high prevalence of not seeking antenatal care services were more likely to experience the services of unskilled birth attendants, those in the southern part with the highest prevalence of low community literacy were also more likely to seek services from unskilled birth attendants. Hence, interventions geared toward reducing and preventing services from unskilled birth attendants but increasing services of skilled birth attendants may focus on the parts of Chad with disadvantaged socioeconomic conditions.

Despite the significant findings revealed in our study, some limitations need to be acknowledged. First, our study is cross-sectional and therefore we were unable to examine the temporal sequence of events to make causal inferences. Also, personal level measures of the women were self-reported, which is susceptible to recall and social desirability biases leading to either under or over-estimation of results. Additionally, we were unable to identify specific districts, towns, and villages in our geospatial analysis to determine the exact location of the women who may be at risk of seeking services from unskilled birth attendants.

Considering that the DHS data did not capture the specific in-depth reasons why unskilled birth attendance is significantly associated with the identified variables in this study, qualitative approaches can be employed in exploring further meanings of these results. For instance, uneducated mothers with low wealth statuses in rural Chad can be interviewed to further understand their unmet needs regarding the utilisation of services from skilled birth attendants.

## Conclusions

Our study shows that more than half of the women in Chad (61.5%) utilised pregnancy and child-delivery services from unskilled birth attendants. We conclude that unskilled birth attendance was highly clustered in all parts of Chad except the southern part owing to various individual and community level factors. Specific individual-level factors associated with the likelihood of using services from unskilled birth attendants in Chad include lower educational level, lower wealth status, lack of adequate access to media messages, having no desire for more births, and lower antenatal care visits during pregnancy. Low community literacy levels and rural residency are significant community-level factors associated with higher odds of utilising services from unskilled birth attendants in Chad. These findings suggest that multiple individual and community-level interventions should be developed to address the high level of unskilled birth attendance in Chad. Improving maternal education and community literacy among women and supporting them to utilise the services of skilled birth attendants during antenatal care can be useful strategies in addressing the problem of unskilled birth attendance in the country.

## Supplementary Information

**Additional file 1.** Moran’s I SpatialAutocorrelation of unskilled birth attendance in Chad.

## Data Availability

Dataset for this study is freely available for download at: http://dhsprogram.com/data/available-datasets.cf

## Abbreviations

AIC: Akaike’s information criterion
AOR: Adjusted Odds Ratio
CI: Confidence Interval
DHS: Demographic and Health Surveys
EA: Enumeration areas
GWR: Geographically weighted regression
ICC: Intra-cluster correlation
LR: Likelihood Ratio
PSU: Primary sampling unit
SSA: Sub-Saharan African
STIs: Sexually transmitted infections
VIF: Variance inflation factor
WHO: World Health Organisation
