# Supplementary figures and images for: Spatial and multilevel analysis of unskilled birth attendance in Chad
Source: BMC Public Health. 2022 Aug 16;22:1561. doi: 10.1186/s12889-022-13972-6 (PMC9382725; doi:10.1186/s12889-022-13972-6)

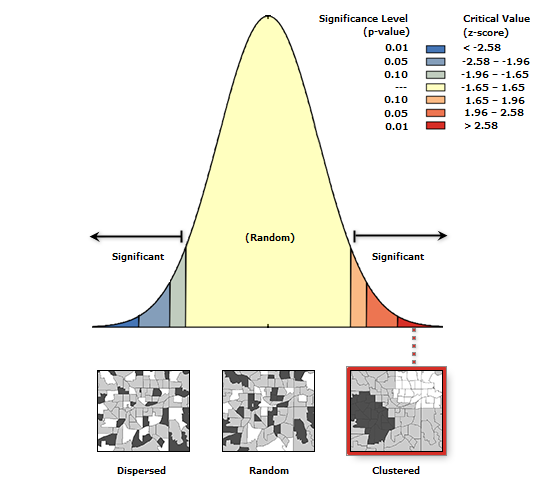


Additional file 1: Moran’s I Spatial Autocorrelation of UBA in Chad

Supplement: Supplementary file 1 — Additional file 1. Moran’s I SpatialAutocorrelation of unskilled birth attendance in Chad. [file 12889_2022_13972_MOESM1_ESM.docx]
